# A New Method for Fractionation and Characterization of Polyphenols and Tannins from Grapevine Leaf Tissue

**DOI:** 10.3390/plants12081706

**Published:** 2023-04-20

**Authors:** Stephan Sommer, Marnelle Salie, Esteban Garcia, Anthony Reyes, Steven C. Ebersole, Rachel P. Naegele, Sonet Van Zyl

**Affiliations:** 1 Grape and Wine Institute, University of Missouri, 223 Eckles Hall, Columbia, MO 65211, USA; 2 Viticulture and Enology Research Center, California State University, 2360 E. Barstow Ave, Fresno, CA 93740, USA; 3 Sugarbeet and Bean Research Unit (SBRU), USDA ARS, 1066 Bogue St. #384, East Lansing, MI 48824, USA

**Keywords:** wine grapes, hydroxycinnamic acids, phenolic profiling, extraction

## Abstract

Plants accumulate different types of phenolic material in their tissue as a response to biotic as well as abiotic stress. Monomeric polyphenols and smaller oligomers can serve as protection against ultraviolet radiation or prevent oxidative tissue damage, while larger molecules such as tannins can be the plant’s reaction to an infection or physical damage. Therefore, characterization, profiling, and quantification of diverse phenolics can provide valuable information about the plant and the stress status at any given time. A method was developed that allows the extraction of polyphenols and tannins from leaf tissue, followed by fractionation and quantification. Extraction was performed with liquid nitrogen and 30% acetate-buffered ethanol. The method was tested with four cultivars under varying extraction conditions (solvent strength and temperature) and showed great improvements of the chromatography that would otherwise be impacted by tannins. The separation of tannins from smaller polyphenols was achieved by bovine serum albumin precipitation and resuspension in a urea-triethanolamine buffer. Tannins were reacted with ferric chloride and analyzed spectrophotometrically. Monomeric non-protein-precipitable polyphenols were then analyzed via HPLC-DAD from the supernatant of the precipitation sample. This way, a more complete spectrum of compounds can be analyzed from the same plant tissue extract. With the fractionation suggested here, hydroxycinnamic acids and flavan-3-ols can be separated and quantified with good accuracy and precision. Possible applications include the assessment of plant stress and response monitoring using the total concentrations of polyphenols and tannins, as well as the ratios between those compound classes.

## 1. Introduction

Phenolic substances play a very important role in plant tissue and serve different purposes depending on their molecular structure and size. Due to the abundance of oxidizable hydroxyl groups, the most common function is stress response [1,2,3], which can be categorized into biotic and abiotic stresses. Even though there are exceptions to that rule, biotic stress most commonly leads to the accumulation of larger polyphenols and tannins [4,5], while abiotic stress such as UV radiation or drought stress triggers the production of smaller functional molecules that can be very specific in nature [1,6]. Characterizing the phenolic profile in plant tissue can therefore provide valuable information about plant status and possible stressors. Hydroxycinnamic acid derivatives, for example, have been shown to protect plant tissue from excess UV radiation and the resulting oxidative damage [7]. If the stress is not too severe, the plant uses these molecules with high antioxidant capacity to capture free radicals that would otherwise lead to the elevated production of reactive oxygen species, resulting in severe and potentially irreparable damage [8]. Other polyphenols such as flavan-3-ols or monolignols, which are derived from hydroxycinnamic acids, are precursors for large polymerized molecules such as tannins or lignin, respectively, which can play a structural or functional role in plants [9,10]. The total concentration in plant tissue and the timing of accumulation are important indicators for the assessment of plant health and stress-coping mechanisms [10,11].

Analyzing phenolic compounds in general can be challenging due to the immense variability in molecule size, reactivity, structure, and composition. While monomeric polyphenols are commonly analyzed by liquid chromatography [12,13,14], there are size limitations for this technique with the quantification of polymeric polyphenols [15,16]. High concentrations of tannins can influence the accuracy and repeatability of the method. The increase in baseline noise, often referred to as the “tannin hump” [16,17], lowers the sensitivity due to the general increase in signal intensity. It is therefore imperative to use a sample preparation that separates small polyphenols from large polyphenols prior to analyzing the fractions. The analysis of tannins is mostly done by spectrophotometric methods that can be based on reactivity with proteins [18,19], polysaccharides [20], or the development of colored compounds [14]. Other destructive methods such as phloroglucinolysis [21,22] provide information about the composition of tannin but fail to identify the structure, size, or reactivity of the molecules. They are therefore less useful for the characterization of the chemical nature of tannin materials. The concept of analyzing polymeric polyphenols and tannins through a colorimetric reaction with iron chloride has been described for wine before [18,19]. Even though the method is unspecific and produces a blue molecule complex with all polyphenols independent of their size, it is sensitive and robust [19].

Methodologies that combine the extraction, analysis, and characterization of monomeric polyphenols and polymeric molecules from the same sample do not exist to the best of our knowledge. Therefore, the objective of this study was to develop a methodology that allows for the separate analysis of monomeric polyphenols as well as tannins from the same plant tissue sample, limiting the use of sample materials and shortening the time.

## 2. Results

**Figure 1:**
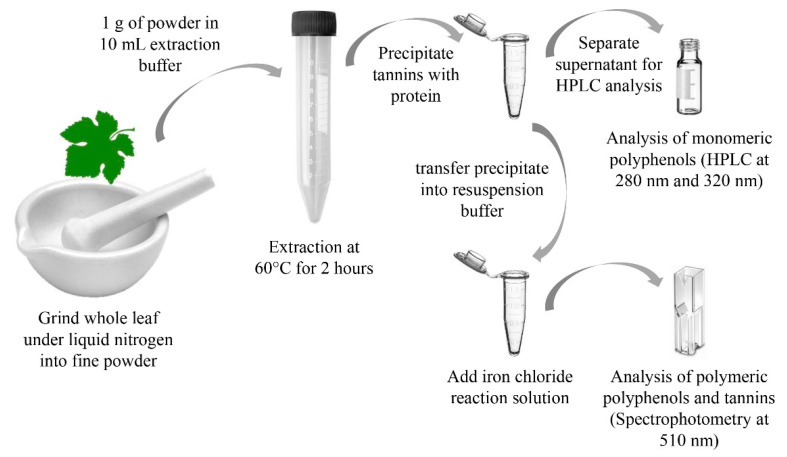
Proposed procedure to extract and separate monomeric and polymeric polyphenols from leaf tissue in grapevines.

Previous extraction methods mostly use a combination of solvents to quantitatively extract phenolics from plant materials [14,23,24]. To minimize chemical use as well as hazardous waste, the proposed procedure uses a combination of physical cell rupture and mild chemical and thermal extraction. The method overview is shown in Figure 1.

The advantages of this procedure can be found in the simplicity regarding hazardous material handling and the analytical optimization through the separation of chemical compound classes that would otherwise affect the sensitivity of the method. The use of liquid nitrogen for the initial sample preparation has been reported before [24,25,26], and it is a very quick step that also protects the sample from excess oxidation due to low temperatures and protective nitrogen gas.

**Figure 2:**
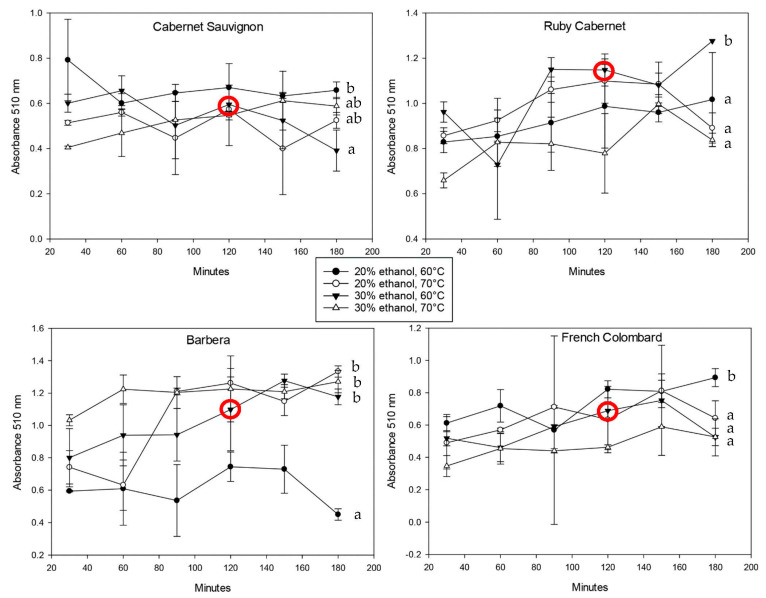
Spectrophotometric analysis of iron-reactive protein-precipitable phenolics in leaf tissue extracts over time. Extracts are shown for four cultivars using different temperature-solvent strength combinations. The red circle indicates the extraction conditions that were chosen for the proposed method. Statistically significant differences (*p* < 0.05) within each dataset are indicated using letters a, b.

In order to achieve a near-exhaustive extraction of the powdered plant material, the two factors, i.e., temperature and solvent strength, were tested on 1 g per 10 mL of extraction buffer suspension. The extraction was monitored every 30 min for a total duration of three hours. Mixing and continuous extraction were ensured by gently shaking the extraction tubes every 30 min. Figure 2 shows the spectrophotometric data of iron-reactive protein-precipitable phenolics in all cultivars over the total extraction time.

**Figure 3:**
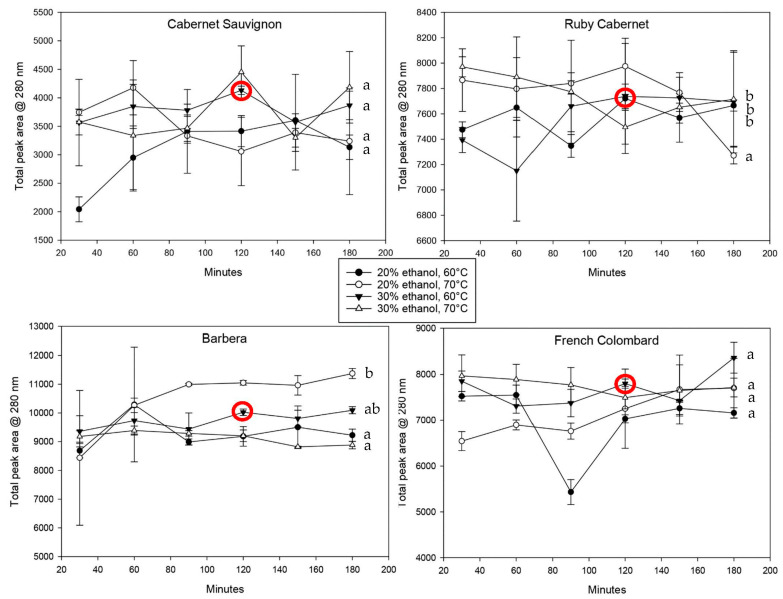
Liquid chromatography analysis of the monomeric phenolic material in leaf tissue extracts over time at 280 nm. Extracts are shown for four cultivars using different temperature-solvent strength combinations. The red circle indicates the extraction conditions that were chosen for the proposed method. Statistically significant differences (*p* < 0.05) within each dataset are indicated using letters a, b.

**Figure 4:**
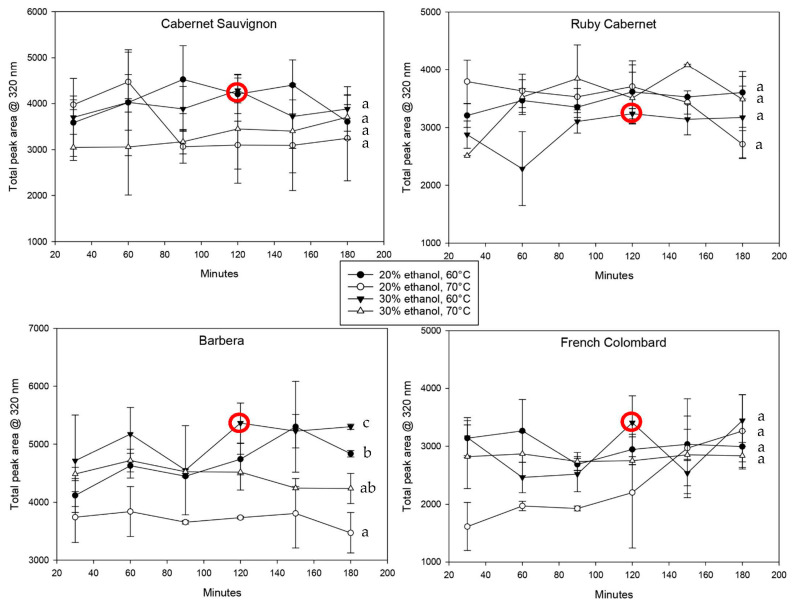
Liquid chromatography analysis of the monomeric phenolic material in leaf tissue extracts over time at 320 nm. Extracts are shown for four cultivars using different temperature-solvent strength combinations. The red circle indicates the extraction conditions that were chosen for the proposed method. Statistically significant differences (*p* < 0.05) within each dataset are indicated using letters a, b, c.

The standard deviation for all trials was relatively large, which can be explained with inconsistencies based on non-linear extraction kinetics. Extractions from a solid material in a liquid are always based on the formation of an equilibrium between the solubilized material and compounds remaining in the solid. Since polyphenols are very reactive regarding oxidation and polymerization, the equilibrium can shift and lead to a temporary loss of free polyphenols followed by re-extraction from the plant material. In addition to that, the extractions shown in Figure 2, Figure 3 and Figure 4 were performed in separate tubes, which means that single extractions were not followed over time but rather conducted independent of each other. This detail and predictable inconsistencies among the ground leaf material might have led to the higher standard deviation. In most cases, however, the concentration of the phenolic material in the extract increased with time. There was no consistent trend regarding solvent strength and temperature, but a lower extraction temperature seemed to yield higher concentrations of phenolics. This can be explained with a better protection against oxidation, since chemical reactions are favored at higher temperatures [27]. Most extractions reached a plateau after two hours, either decreasing or staying constant after that. The decrease could be due to oxidative loss of phenolic compounds as well as precipitation reactions with the cell wall material such as proteins and polysaccharides. It is important to note that the molecules that were included in the protein-precipitable iron-reactive phenolics are polymeric in nature, resulting in less oxidative loss but a higher degree of hydrophobicity that generally leads to more precipitation [28].

Monomeric phenolic compounds that were smaller in size were evaluated by high-performance liquid chromatography (HPLC) at 280 nm and 320 nm, representing all polyphenols and hydroxycinnamic acids (HCAs), respectively. Figure 3 shows the total peak areas at 280 nm for all cultivars and extraction conditions.

As previously observed, most extractions followed a time-dependent pattern; however, the increase over time was generally lower than for polymeric polyphenols. The reason for this can be hypothesized to be due to the more hydrophilic nature of small molecules. Overall extraction and the saturation equilibrium will be reached much sooner with small molecules, but they are also more sensitive to oxidation due to their higher degree of reactivity. Especially the higher temperatures tend to show a decrease in concentrations, most likely caused by oxidative loss.

The hydroxycinnamic acids, shown in Figure 4, confirm this observation. Most extractions reached their maximum concentration at the first sampling point after 30 min and changed relatively little after that. Interestingly, while the extraction with 20% ethanol at 70 °C yielded good results for total polyphenols (Figure 3), it showed the lowest extraction for most of the cultivars for hydroxycinnamic acids. It remains unclear why the two groups of compounds behave inconsistently, but it can be hypothesized that HCAs are easier to oxidize based on their molecular structure [29] and should therefore not be extracted at higher temperatures [27].

Comparing the cultivars directly regarding phenolic extraction, it becomes obvious that the variability with all polyphenols was much higher compared to that with hydroxycinnamic acids. Barbera leaves, for example, contained twice as much phenolic material as Cabernet Sauvignon leaves (Figure 3) but were not much different when looking at HCAs specifically. However, for the purpose of this method development, hydroxycinnamic acids are the more important class of compounds due to their ability to mediate biotic and abiotic stress [1,6]. The conditions to achieve the maximum extraction were therefore weighted in favor of the results obtained by HPLC at 320 nm.

**Figure 5:**
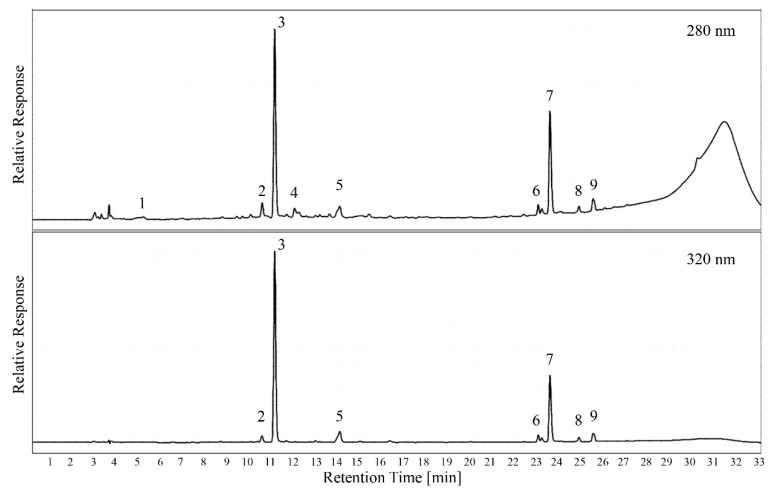
Example chromatogram of the phenolic material in leaf tissue extracts at 280 nm and 320 nm. Peak identification was performed with pure standards and a spectral comparison with a database (1: gallic acid; 2: catechin; 3: caftaric acid; 4: epicatechin; 5: caffeic acid; 6: unidentified hydroxycinnamic acid; 7: caffeic acid derivative; 8: coumaric acid derivative; 9: ferulic acid derivative).

Figure 5 shows a set of sample chromatograms including the peak identification that was performed with pure standards and spectral information. While previous studies have shown a large number of different phenolic compounds in grape berries [30], leaves showed relatively little variety in monomeric phenolics. It is also interesting to note that the number of unique peaks at 280 nm was very low. Since all HCAs that absorbed at 320 nm also absorbed at 280 nm [12], the concentration of small non-HCA phenolics varied more among grape cultivars than under extraction conditions.

Even though previous studies have identified over 100 different phenolic compounds in grapevine plant tissue [13], the number of analytes that exceed the trace level is relatively small. The polyphenols identified in this study are in agreement with compounds that were previously reported in grapevine leaves [13]. As expected, caffeic acid derivatives such as caftaric acid account for the majority of small polyphenols in leaf tissue. The separation of peaks and the chromatography conditions, however, allow for quantification of all major hydroxycinnamic acids and flavanols, even if they do not appear in the sample chromatogram in Figure 5. Interestingly, the chromatogram recorded at 280 nm still showed a “tannin hump” after 28 min, even though the tannins were previously separated using protein precipitation. However, the baseline in the area where peaks were quantified was not affected by this, and there was no carry-over between chromatography runs. The hump was also not visible at 320 nm, indicating that it represents oligomers of polyphenols that only absorb at 280 nm.

## 3. Discussion

The simultaneous extraction and separation of small and large polyphenolic molecules can be achieved from the same sample with minimal additional preparation. The methods that were combined and modified here have been validated for grape berries, juice, and wine [18,19,31] but have not been described for leaf extracts before. Using liquid nitrogen for the initial grinding offers multiple advantages over other techniques. The most important aspect is probably that it can be performed with fresh, frozen, or dried leaves. Liquid nitrogen freeze-dries the leaf material almost instantly and allows for physical force to create a fine powder that is also protected from oxidation. The ability to process leaves right after collection in the vineyard or store them prior to processing enables the maximum flexibility during the growing season.

Traditional methods for analyzing polyphenols often use solvents for a quantitative extraction from the sample material [24,27,32]. The advantage of a quantitative extraction is that all classes of compounds as well as all molecule sizes can be analyzed in the extract. Disadvantages are related to the diversity of chemical properties and the analytical interferences that can result from that. Analyzing smaller-size polyphenols in the presence of large tannins, for example, results in a loss of sensitivity, when it is performed in the undiluted extract using HPLC. The polymeric polyphenols produced a “tannin hump” (Figure 5) that was caused by molecules that only had a loose interaction with the stationary phase and constantly eluded from the column over a longer time in the chromatogram. The resulting elevated absorbance reading lowered the sensitivity for compound peaks that sat on top of the “tannin hump”, making an accurate quantification more challenging. The method proposed here addresses that problem in two different ways.

Instead of a quantitative extraction, ethanol at elevated temperatures in combination with a buffer creates an extraction equilibrium. This has been shown for different extraction processes and matrices before [14,33]. Ethanol in relatively low concentrations improves the solubility of more hydrophobic molecules such as tannins [34] without suppressing the solubility of smaller polyphenols and phenolic acids that are more hydrophilic in nature. By adjusting the ethanol concentration to the right solvent strength, an equilibrium is created that is representative of the ratios that could be achieved by a quantitative extraction [14]. At the same time, ethanol is a mild solvent which does not create hazardous waste during the process [30] and can be injected into an HPLC system without compromising the chromatography which could happen in the case of acetone for example. Ethanol as a short-chain alcohol also avoids the overextraction of hydrophobic molecules that could cause interferences with the resuspension step during the spectrophotometric analysis of iron reactive phenolics.

Based on the observation that there were very few statistically significant differences between extractions with 20% or 30% ethanol, the final method was proposed to use a 30% solvent strength. A higher ethanol concentration results in a more complete extraction of molecules that are more hydrophobic in nature. This also includes cell wall polysaccharides and structural proteins [35]; however, the use of iron chloride for the quantification allows for good selectivity and only creates a colored complex with polyphenols. There were no expected interferences based on the original validation that was performed in a red wine matrix. While higher temperatures during the extraction process can provide better cell rupture and higher extraction rates, temperatures above 70 °C lead to a faster degradation of polyphenolic materials [27]. For that reason and the aforementioned lower extraction and retention of hydroxycinnamic acids at 70 °C, the temperature of the final method was set to 60 °C. On average, the extraction plateau at that temperature was reached after 114 min with a standard deviation of 42 min. Even though the variability among all grape cultivars tested was relatively large, the optimum extraction time was set to 120 min. This allowed enough time to reach an extraction equilibrium while minimizing the risk of oxidative loss due to excessive heat and time.

The separation of tannins from the extract by means of protein precipitation was adapted from a method that is commonly used in red wine [18,19]. It is based on the assumption that larger polyphenols form insoluble complexes with bovine serum albumin due to their degree of hydrophobicity. Those tannin-protein agglomerates can be separated by centrifugation and resuspended in a urea buffer for analysis. It would be an oversimplification to assume that this separation is solely based on molecule size; however, protein-precipitable polyphenols are generally larger molecules [36], but not all larger polyphenols can react with proteins. We introduced this separation step to allow for better sensitivity during HPLC analysis, but a portion of polymeric polyphenols that were not protein-precipitable were still visible in the chromatogram (Figure 5). This fraction was eluted from the column at high acetonitrile concentrations and occurred after the peaks of interest; however, it represents a portion of polyphenols that cannot be quantified with the current method. This third fraction in addition to protein-precipitable tannins and monomeric polyphenols might not be of physiological interest for the grapevine, but it requires further clarification and characterization.

## 4. Materials and Methods

### 4.1. Selection of Leaf Tissue

In August 2021, five to seven mature leaves were collected from Cabernet Sauvignon, Ruby Cabernet, Barbera, and French Colombard, depending on the cultivar-specific size of the undamaged leaves. All vines were grown on the University Agricultural Laboratory Farm at California State University in Fresno and were fully mature and managed as a commercial vineyard. Leaves were collected from different locations on the vine but were all sun-exposed and free of any visible disease, damage, or spray residue. This ensured that the number of variables that could influence the concentration of tannins and polyphenols was limited to the cultivar and the growing conditions, while minimizing variability within the dataset that could be caused by biotic factors. All leaves from one cultivar were sealed in a Ziplock bag and stored at −80 °C for processing and analysis.

### 4.2. Extraction Conditions

All leaves of each cultivar were processed as one batch in order to create a uniform sample from the different locations on the vine. All leaves were cut into smaller pieces, directly after they were removed from the freezer. The material was placed in a mortar, submerged in liquid nitrogen and ground into a fine powder using a pestle. The uniform powder was then split into 1 g samples and divided into 24 15 mL centrifuge tubes.

The extraction buffer was created with two different ethanol concentrations (20% *v/v* and 30% *v/v* in water) to evaluate the effect of solvent strength. Then, 9.86 g/L sodium chloride were dissolved in the ethanol solution, 12 mL/L glacial acetic acid was added, and the pH was adjusted to 4.9.

To each of the previously prepared 15 mL centrifuge tubes containing 1 g of powdered sample, we added 10 mL of the extraction buffer. Half of the tubes (12) contained 20% ethanol, while the other half of the tubes were extracted with 30% ethanol. The tubes were then placed in a temperature-controlled water bath for 3 h, following the specific sampling protocol that is outlined in Table 1. All extractions were performed in duplicates.

Each sample that was removed from the water bath was centrifuged at 6000 RPM (3750 RCF) for 10 min and decanted into a separate reaction tube. The extract was then rapidly cooled to room temperature for further analysis to limit oxidation.

### 4.3. Analysis of Phenolic Compounds

The analysis of protein-precipitable iron-reactive phenolics was adapted from Harbertson et al. [18,19], and the liquid chromatography method for the quantification of monomeric polyphenols was adapted from Sommer et al. [31]. Briefly, 200 µL of the leaf material extract was added to 1000 µL of a bovine serum albumin (BSA) tartrate buffer (1 g/L BSA, 2.5 g/L potassium bitartrate in 12% ethanol, pH 3.6) and incubated for 15 min. The sample was centrifuged in an Eppendorf tube at 10,000 RPM for 5 min, and the supernatant was transferred into an HPLC vial for further analysis. The precipitate was resuspended in 800 µL of urea buffer (498 g urea in 800 mL water with 50 mL triethanolamine, adjusted to pH 8.0 and filled up to 1000 mL with water), followed by the addition of 125 µL of ferric chloride solution (2.7 g ferric chloride in 800 mL water, 800 µL HCl added and filled to 1000 mL with water). The mixture was vortexed, until the precipitate was fully dissolved and incubated for 10 min. The samples were then decanted into a 1.5-mL cuvette and analyzed at 510 nm against a reagent blank (125 µL ferric chloride solution with 875 µL urea buffer).

For the liquid chromatography analysis, an Infinity II 1260 HPLC-DAD instrument (Agilent Technologies Inc., Folsom, CA, USA) was used. The column was an InfinityLab Poroshell 120 EC-C18 with the dimensions of 4.6 × 150 mm and a particle size of 4 µm (Agilent Technologies Inc., Folsom, CA, USA). Mobile phase conditions started at 100% mobile phase A (10 mM KH_2_PO_4_/H_3_PO_4_ buffer at pH 1.5 in 5% acetonitrile in water; mobile phase B: 10 mM KH_2_PO_4_/H_3_PO_4_ buffer at pH 1.5 in 50% acetonitrile in water) and decreased to 85% by 7 min, from 85% to 70% after 15 min, from 70% to 60% by 20 min, from 60% to 40% by 24 min, and to 10% after 27 min. After 30 min, it was set back to initial conditions (100% mobile phase A) for 3 min to reach equilibrium (total analysis time: 33 min). The detection wavelengths were set to 280 nm and 320 nm. Various pure standards (all purchased from VWR, Radnor, PA, USA) were used to verify retention times and quantify selected compounds through an external calibration. All quantifiable peaks with an absorbance at 280 nm were also summarized as total flavanols and expressed as catechin equivalents. All quantifiable peaks at 320 nm were summarized as total hydroxycinnamates and calculated as caffeic acid equivalents.

### 4.4. Statistical Analyses

Data handling and statistical analysis were conducted using SigmaPlot 14.0 (Systat Software Inc., San Jose, CA, USA). Experiments were performed in duplicates, and the data were analyzed using a one-way ANOVA with post hoc Tukey’s test for pairwise comparison.

## 5. Conclusions

A new method was developed that selectively extracts and separates polymeric protein-precipitable polyphenols from smaller molecules and analyzes both groups separately from the same plant tissue sample. The colorimetric method for larger polyphenols was adapted from red wine analysis and has a high degree of specificity and no known interferences. The separation of these larger compounds from the extract allows for a more sensitive analysis of monomeric polyphenols using HPLC. The advantages of the new method are the low usage of solvents and reagents, the short sample preparation and analysis time, as well as the quantitative nature of the analyses. The method can be used to analyze physiological markers for biotic and abiotic stress in plant tissue and quantify the impacts of specific stressors.

Future research is needed to characterize non-protein-precipitable polymeric polyphenols that cause a baseline increase during HPLC analysis. It does not interfere with the method presented here, but it remains unknown if that fraction has any physiological importance for the plant for either biotic or abiotic stress response.

## Figures and Tables

**Table 1:** Extraction conditions and sampling protocol to identify the preferred solvent strength, temperature, and time of extraction.

Solvent Strength	Temperature	Sampling
20% ethanol (*v/v*)	60 °C	6 tubes total of each cultivar for each extraction condition; shaking every 30 min; 1 tube removed for analysis every 30 min; total maximum extraction time: 3 h
20% ethanol (*v/v*)	70 °C	
30% ethanol (*v/v*)	60 °C	
30% ethanol (*v/v*)	70 °C	

## Data Availability

All data is in the manuscript.
